# Application of Visible and Near Infrared Spectroscopy for Rapid Analysis of Chrysin and Galangin in Chinese Propolis

**DOI:** 10.3390/s130810539

**Published:** 2013-08-13

**Authors:** Pengcheng Nie, Zhengyan Xia, Da-Wen Sun, Yong He

**Affiliations:** 1 College of Biosystems Engineering and Food Science, Zhejiang University, Hangzhou 310058, China; E-Mail: npc2012@zju.edu.cn; 2 Cyrus Tang Center for Sensor Materials and Applications, Zhejiang University, Hangzhou 310058, China; 3 Zhejiang Research Institute of Traditional Chinese Medicine, Hangzhou 310023, China; E-Mail: xzhengyan@gmail.com; 4 Food Refrigeration and Computerised Food Technology (FRCFT), Agriculture & Food Science Centre, School of Biosystems Engineering, University College Dublin, National University of Ireland, Belfield, Dublin 4, Ireland; E-Mail: dawen.sun@ucd.ie

**Keywords:** Chinese propolis, chrysin, galangin, visible and near infrared spectroscopy (Vis-NIR), successive projection algorithm, partial least squares, back propagation-artificial neural networks

## Abstract

A novel method for the rapid determination of chrysin and galangin in Chinese propolis of poplar origin by means of visible and near infrared spectroscopy (Vis-NIR) was developed. Spectral data of 114 Chinese propolis samples were acquired in the 325 to 1,075 nm wavelength range using a Vis-NIR spectroradiometer. The reference values of chrysin and galangin of the samples were determined by high performance liquid chromatography (HPLC). Partial least squares (PLS) models were established using the spectra analyzed by different preprocessing methods. The effective wavelengths were selected by successive projections algorithm (SPA) and employed as the inputs of PLS, back propagation-artificial neural networks (BP-ANN), multiple linear regression (MLR) and least square-support vector machine (LS-SVM) models. The best results were achieved by SPA-BP-ANN models established with the Savitzky-Golay smoothing (SG) preprocessed spectra, where the r and RMSEP were 0.9823 and 1.5239 for galangin determination and 0.9668 and 2.4841 for chrysin determination, respectively. The results show that Vis-NIR demosntrates powerful capability for the rapid determination of chrysin and galangin contents in Chinese propolis.

## 1. Introduction

Propolis, also called bee glue, is a brownish, sticky resinous substance collected by honeybees from leaf buds and cracks in the bark of certain trees and plants. Propolis has a complex chemical composition and has been used widely in folk medicine for many years. It is reported that propolis has many biological and pharmacological characteristics, including antibacterial, anti-inflammatory, antiviral, antitumor, anticancer, and immunomodulatory effects [1]. There are over 150 constituents in propolis, including polyphenols, terpenoids, steroids and amino acids. Flavonoids, as one of the most important groups, can represent around 50% of the propolis contents, depending on the harvest region, since the characteristics of propolis are influenced by the local plant varieties and weather [2]. Chrysin and galangin as two of the main flavonoids in propiolis are generally analyzed using chromatographic methods according to the Chinese Pharmacopeia and the current standard of Ministry of Agriculture of China [3]. Generally, methods for determining chrysin and galangin in Chinese propolis include TLC, GC [4] and HPLC [5,6], that are helpful in identification and quantification of the various chemical constituents of propolis, but these methods are complex and time-consuming. Therefore, it is necessary to develop a rapid and effective quantitative analysis method for the quality determination of Chinese propolis.

With the development of spectroscopic techniques and modern chemometrics, visible and near infrared (Vis-NIR) spectroscopy that is considered to be non-destructive, simple and rapid, has been widely applied in the research of agricultural, food, and natural products [7–10]. Especially, in recent years, there are many reports about the application of spectroscopy techniques in various aspects of research on traditional Chinese medicines (TCMs), such as geographical source identification, quality control, stability forecasting, *etc.* [11–14]. However, there are few studies evaluating the potential of Vis-NIR for quantitative analysis of chrysin and galangin in Chinese propolis.

The objective of the study was thus to develop a new method to quantitatively and non-destructively determine the contents of chrysin and galangin in Chinese propolis by the Vis-NIR technique. For this purpose the performances of established prediction models using different chemometric methods were compared and evaluated.

## 2. Materials and Methods

### 2.1. Apparatus and Reagents

ASD FieldSpec Pro FR (350–1,075 nm, Analytical Spectral Device, Boulder, CO, USA), Agilent 1100 high performance liquid chromatograph (Agilent Technologies Inc., Santa Clara, CA, USA), KQ-100DB ultrasonic cleaner (Shanghai, China), Mettler Toledo AB204-S electronic balance (Zurich, Switzerland).

Chrysin and galangin were purchased from the National Institute for the Control of Pharmaceutical and Biological Products (Beijing, China). The HPLC-grade methanol and acetonitrile both were obtained from Tedia Scientific Inc. (Cincinnati, OH, USA). Phosphoric acid (analytical grade, P85%) was purchased from Zhejiang Chemicals Company (Zhejiang, China). All the other reagents were of analytical grade. Water used throughout the experiments was purified water provided by Wahaha Company (Zhejiang, China).

A total of 114 samples of Chinese propolis of poplar origin used in this research were purchased from beekeepers in the Shandong, Jilin, Anhui, Zhejiang, Jiangsu, Jiangxi and Henan provinces of China. Each sample was dehydrated into a powder. Among the prepared samples, 76 samples were selected randomly to be used as the calibration set, and the remaining 38 samples were used as the prediction set.

### 2.2. Spectra Measurements

Each sample was put in a Petri dish and then scanned using a spectroradiometer working in the wavelength range of 325 to 1,075 nm. A white disk was used as the reference board. Spectra data were collected and processed using RS^2^ V4.02 software for Windows (Analytical Spectral Devices, Inc., Boulder, CO, USA). The probe of the spectroradiometer was fixed 100 mm above the surface of the sample with the field of view (FOV) of 25° and an angle of 45° away from the center of the sample container. Each sample was scanned 30 times, and the acquired spectra were averaged as the measured spectrum of this sample.

### 2.3. Liquid Chromatographic Conditions

Contents of chrysin and galangin were determined on an Agilent 1100 series HPLC system, which consists of a G1322A vacuum degasser, a G1311A quaternary pump, a G1329A autosampler, a G1314B programmable variable wavelength detector (VWD), and a G1316A Thermostatted Column Compartment. All analyses were performed by using a Diamonsil C_18_ column (250 × 4.6 mm, 5 μm) at 30 °C. The detection wavelength was set at 268 nm. The mobile phase consisted of (A) methanol and (B) 0.15% aqueous phosphoric acid at a flow rate of 1 ml/min. Separations were performed by the following linear gradient: 64% A in 25 min, 75% A in 8 min. The injection volume was 10 μL.

### 2.4. Pretreatment of Spectral Data

Before the calibration process, the spectra of all samples were pretreated to reduce baseline variation, light scattering, and path length differences using several preprocessing algorithms, including Savitzky-Golay smoothing (SG), moving averages smoothing (MAS), standard normal variate transformation (SNV), multiplicative scattering correction (MSC), the first derivative (1st-Der), the second derivative (2nd-Der) and de-trending (De-trending). The details of these pretreatment methods could be found in the literature [15]. These methods were compared to choose the optimum preprocessing strategy. The pre-process and calculations were carried out using the Unscrambler X10.1 software (Camo Process AS, Oslo, Norway).

### 2.5. Data Analysis

Partial least square (PLS) [16] was applied to develop the calibration models as well as a way to extract latent variables (LVs). PLS is performed to establish a regression model to perform the prediction of physiological concentrations [17]. The LVs are considered as new eigenvectors of the original spectra to reduce the dimensionality and compress the original spectral data.

Multiple linear regression (MLR) is aimed to establish a direct, simple, and linear combination of independent variables (referring spectral wavelengths in this work, *X*) that corresponds as closely as possible to the dependent variable (referring a quality attribute, *Y*) [18,19]. The drawback of MLR is that the number of samples for MLR must be larger than the number of variables. In this study, effective wavelengths (EWs) were set as the independent variables of MLR, so that the number of input variables of the MLR model could be smaller than that of samples.

A back propagation-artificial neural network (BP-ANN) as one of the most popular neural network topologies, was employed in this paper to establish the relationship between EWs and galangin/chrysin contents. In the calculation of BP-ANN, the EWs are introduced into the network as inputs via the nodes of the input layer. The input signals are then transferred from the input node to the output node via the hidden layer. The BP-ANN model is developed by adjusting the nodes of hidden layers and other parameters.

Least square-support vector machine (LS-SVM) is a modified algorithm based on the classical SVM and has been applied for spectral analysis [20]. It uses a set of least squares linear equations as loss functions instead of the quadratic programming to obtain the supported vectors, and is capable of dealing with linear and nonlinear multivariate calibration and solves multivariate calibration problems in a relatively fast way [21]. It is very important to select a proper kernel function and determine its optimal parameters for the construction of LS-SVM models. Radial basis function (RBF) is a simple Gaussian function that can simplify the complexity of the computation during the course of training LS-SVM models, so that RBF kernel was chosen for LS-SVM modeling in the study. It was found having more capability in prediction than other kernels [22]. The formula of RBF can be expressed as:

(1) $$\left[f(x)=\sum_{i=1}^{n}\alpha_{i}K(x,x_{i})+b\right]$$

where *K(x*, *x_i_)* is the kernel function, *x_i_* is the input vector, *α_i_* is a Lagrange multiplier, while b is the bias term. The optimal parameter values of the regularization parameter (gam(γ)) and the RBF kernel function parameter (sig^2^(σ^2^)) were determined according to the smallest root-mean-square error of cross-validation (RMSECV) [23].

In this study, EWs were selected in order to reduce the input variables and improve the speed of model calibration. Successive projection algorithm (SPA) was used to identify EWs from the whole spectral range, where a variable group that contains a minimum of redundant information in the spectral matrix is chosen to minimize the colinearity of different variables, so that the input variables were simplified and the efficiency of modeling could be improved. SPA was performed by Matlab 7.10.0 software (The Mathworks, Inc., Natick, MA, USA). EWs selected by SPA were employed as the inputs of PLS, BP-ANN, MLR and LS-SVM to develop calibration models, and their performances were compared. The correlation coefficient (r) and root mean square error of prediction (RMSEP) were applied to evaluate the performances of the established models. PLS and MLR were implemented by Unscrambler X10.1, and LS-SVM and BP-ANN were compiled by the Matlab 7.10.0 software.

## 3. Results and Discussion

### 3.1. Features of Vis-NIR Spectra and HPLC Analysis

**Figure 1.**
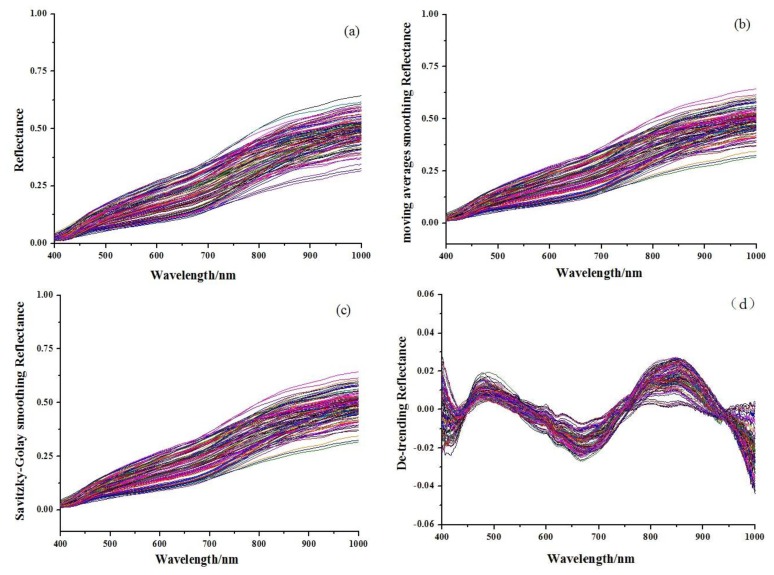
(**a**) Original spectra of Chinese propolis. (**b**) Preprocessed spectra by moving averages smoothing (MAS). (**c**) Preprocessed spectra by Savitzky-Golay smoothing (SG). (**d**) Preprocessed spectra by De-trending.

**Figure 2.**
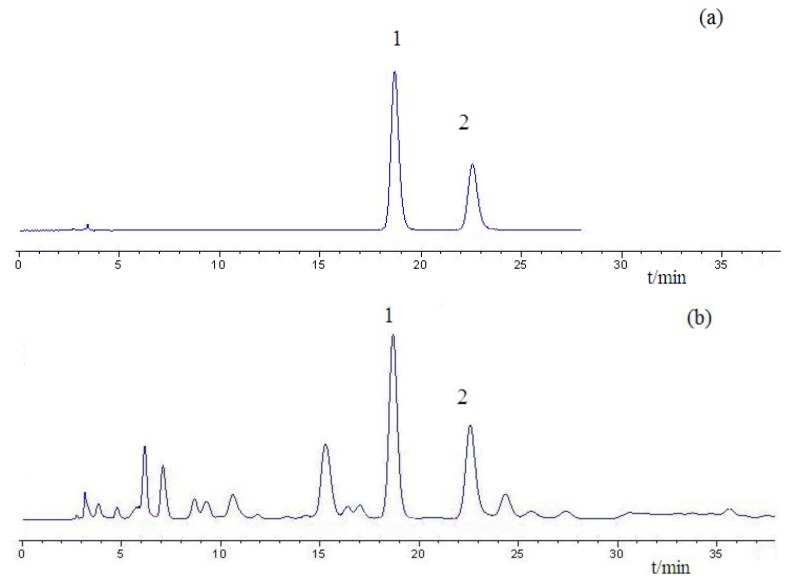
HPLC chromatograms of (**a**) standard solution and (**b**) propolis sample from Henan (1. chrysin; 2. galangin)

Figure 1 shows the original absorbance spectra and preprocessed spectra of 114 Chinese propolis samples. It was noticed that all spectra had similar profiles that were quite even throughout the whole wavelength range. The main difference of spectra was the different magnitudes of the spectral reflectance as shown in Figure 1, which might be caused by different contents of the internal attributes for the samples, including galangin and chrysin. The reference values of chrysin and galangin in Chinese propolis samples determined by HPLC are shown in Table 1. HPLC chromatograms of a typical propolis samples and standard solution are shown in Figure 2.

### 3.2. PLS Analysis

Different spectral pretreatment algorithms were executed on the raw Vis-NIR spectral data. The pretreated spectra were set as inputs to develop PLS models to determine the optimal pretreatment way. Results of the PLS models established using the raw and pretreated spectra are shown in Table 2. The best result was obtained based on De-trending process for the chrysin prediction, followed by SG process. The prediction result of De-trending model had a good correlation coefficient (r) of 0.9476 and a small root mean square error of prediction (RMSEP) of 3.0172. On the other hand, the best PLS model (r = 0.9394 and RMSEP = 2.5733) was achieved by considering the raw spectra for galangin analysis, followed by MAS and SG process. The original/pretreated spectra shown the best performances were employed for further treatment.

### 3.3. EWs Extracted by SPA

SPA was applied to select EWs based on the spectral data processed by raw spectra for galangin, De-trending and SG for chrysin, respectively. These preprocessing methods achieved good prediction performance in PLS models for galangin or chrysin prediction. In addition, the original spectral data were also applied for a comparison. Maximum number of EWs extracted by SPA was set at 30.

After the process of leave-one-out cross validation, the extracted EWs based on the original and pretreated spectra are shown in Table 3. The EWs were arranged according to the importance. Higher ranking indicates the EW is more important for the prediction of chrysin and galangin in Chinese propolis.

### 3.4. Model Calibration

In order to establish quantitative models for the determination of chrysin and galangin in Chinese propolis, EWs selected by SPA were employed as inputs of PLS, LS-SVM, MLR and BP-ANN methods, resulting in establishing SPA-PLS, SPA-LS-SVM, SPA-MLR, SPA-BP-ANN models, respectively (Table 4). In the establishment of SPA-LS-SVM model, the values of two parameters of gam(γ) and sig^2^(σ^2^) were determined by a two-step grid search method with leave-one-out cross-validation. The search region of γ, σ^2^ was set as 10^−3^–10^8^. BP-ANN model was constructed with three layers, and the number of nodes in the hidden layer was set as nine. The least learning rate was set as 0.6, and the goal error was set to 1.0 × 10^−5^. The networks were trained by gradient descend method and the minimum gradient was set as 1.0 × 10^−10^. The biggest epochs was set as 1,000.

**Figure 3.**
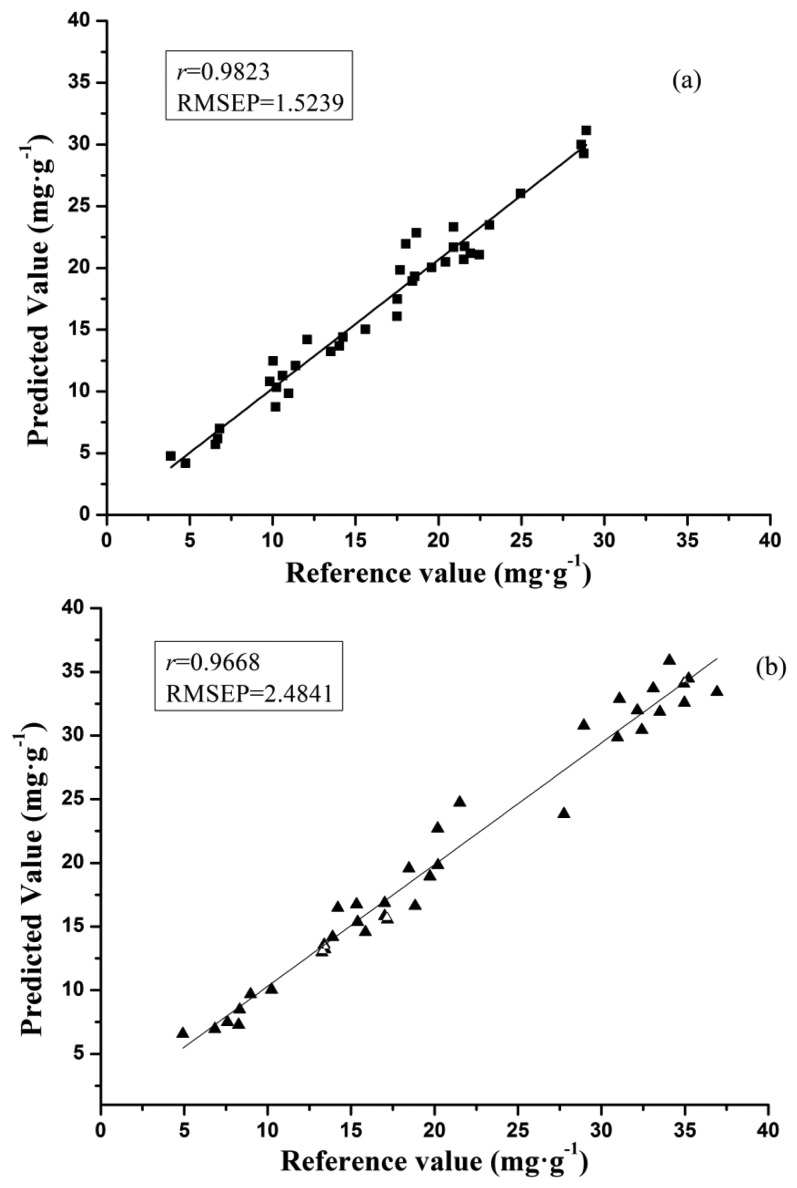
Prediction results of (**a**) galangin and (**b**) chrysin by SPA-BP-ANN models (SG) for samples in the prediction set.

As shown in Table 4, the optimal prediction performance for the galangin determination was achieved by SPA-BP-ANN model (processed by SG) with r of 0.9823 and RMSEP of 1.5239, and SPA-BP-ANN model (processed by SG) was found to be the best one for the chrysin determination, in which r was 0.9668 and RMSEP was 2.4841. The scatter plots of SPA-BP-ANN models for samples in the prediction set in both galangin and chrysin analysis are shown in Figure 3.

As the SPA-BP-ANN model outperformed the SPA-PLS, SPA-MLR and SPA-LS-SVM models, it might indicate that there was a nonlinear relationship between the spectral data and the dependent variable (galangin or chrysin). Therefore, BP-ANN models could perform better prediction by taking useful nonlinear information in the selected EWs, while PLS and MLR models only have the ability to quantify the information in the spectral data to the dependent variable in linear ways.

## 4. Conclusions

In this study, the use of Vis-NIR spectroscopy combined with the reference HPLC method to determine chrysin and galangin contents in Chinese propolis was evaluated. Different pretreatment and modeling methods were compared. In specific, De-trending were determined as the optimal preprocessing method for chrysin, and raw data was the best for galangin. EWs extracted by SPA were proved to be informative inputs for developing models. The best prediction performance with r of 0.9823 and RMSEP of 1.5239 was achieved by the SPA-BP-ANN model for galangin, while SPA-BP-ANN model also had a better performance for chrysin with r of 0.9668 and RMSEP of 2.4841. The results indicate the feasibility of using Vis-NIR spectroscopy with a BP-ANN model chemometrics method based on EWs identified by SPA as inputs to measure chrysin and galangin in Chinese propolis rapidly and quantitatively. In the future, more samples and varieties of propolis should be considered to establish a more stable model for industrial application. In general, a high precision detection of chrysin and galangin was attained by the HPLC method, however, the method was complex and time-consuming. Rapid, nondestructive and efficient determination of chrysin and galangin was achieved by Vis-NIR spectroscopy, although the precision and adaptability still need to be further improved.

## Figures and Tables

**Table 1.** Chrysin and galangin in Chinese propolis determined by HPLC.

**Data Set**	**Sample No.**	**Galangin**			**Chrysin**		
	
		**Range(mg/g)**	**Mean(mg/g)**	**S.D.**	**Range(mg/g)**	**Mean(mg/g)**	**S.D.**
Calibration	76	4.2–34.8	17.2	7.56	6.9-37.3	20.8	9.53
Validation	38	4.2–32.6	17.2	7.53	7.5-34.7	20.8	9.59

S.D.: Standard deviation.

**Table 2.** Results of PLS models with different data pretreatment methods.

**Quality**	**Pretreatment**	**Number of Latent Variables**	**Calibration**		**Validation**		**Prediction**	
		
			***r***	**RMSEC**	***r***	**RMSEV**	***r***	**RMSEP**
Galangin	None	7	0.9437	2.4835	0.9152	3.0366	0.9394	2.5733
	MAS	7	0.9427	2.5053	0.9146	3.0440	0.9370	2.6183
	SG	7	0.9425	2.5092	0.9142	3.0520	0.9360	2.6366
	Normalize	10	0.9751	1.6662	0.9155	3.0547	0.9054	3.1586
	SNV	10	0.9721	1.7608	0.9191	2.9810	0.9057	3.1548
	MSC	9	0.9674	1.8995	0.9175	2.9985	0.9071	3.1286
	1-Der	6	0.9559	2.2049	0.9190	2.9638	0.9307	2.7945
	2-Der	2	0.9269	2.8177	0.8408	4.0652	0.8212	4.2476
	De-trending	9	0.9644	1.9853	0.8936	3.3770	0.9232	2.9015

Chrysin	None	11	0.9877	1.1416	0.9549	2.8303	0.9288	3.6754
	MAS	11	0.9737	1.5342	0.9235	2.6527	0.9282	3.7202
	SG	11	0.9789	1.9397	0.9473	3.0715	0.9474	3.0385
	SNV	5	0.9797	1.9001	0.9393	3.2831	0.9456	3.1314
	MSC	10	0.9228	3.6547	0.8918	4.2966	0.9111	3.9628
	1-Der	8	0.9764	2.0466	0.9388	3.3112	0.9398	3.2202
	2-Der	7	0.9793	1.9198	0.8127	5.5377	0.7643	6.1602
	De-trending	8	0.9722	2.2222	0.9326	3.4352	0.9476	3.0172

**Table 3.** Selected effective wavelengths (EWs) by SPA.

**Quality**	**Pretreatment**	**No.**	**Selected EWs/nm**
Galangin	raw	8	973, 932, 997, 714, 447, 992, 1000, 646
	MAS	13	456, 929, 487, 598, 543, 887, 434, 839, 694, 998, 1,000, 407, 409
	SG	14	931, 456, 486, 600, 542, 886, 698, 995, 434, 839, 994, 997, 998, 412

Chrysin	raw	5	999, 406, 400, 421, 463
	De-trending	9	681, 572, 424, 962, 929, 970, 545, 938, 400
	SG	19	574, 636, 772, 527, 720,849, 443, 886, 430, 460,976, 543, 968, 494, 986, 997, 998, 424 ,409

**Table 4.** Prediction results of considering different pretreatments and calibration methods based on spectroscopy technique for galangin and chrysin analysis.

**Quality**	**Model**	**Pretreatment**	**LV/EW/(γ, σ**^2^**)**	**Prediction**	

				***R****_p_*	**RMSEP**
Galangin	SPA-PLS	Raw	5/8/-	0.8823	3.6368
		MAS	10/13/-	0.9389	2.6105
		SG	10/14/-	0.9387	2.5683

	SPA-LS-SVM	Raw	-/8/(4.62 × 10^3^,0.0042)	0.4000	6.9668
		MAS	-/13/(0.4796,0.0308)	0.4708	7.1444
		SG	-/14/(0.0858, 4.62 × 10^3^)	0.7016	7.3476

	SPA-MLR	Raw	-/8/-	0.8736	3.8170
		MAS	-/13/-	0.9294	2.7915
		SG	-/14/-	0.9505	2.3154

	SPA-BP-ANN	Raw	-/8/-	0.9269	3.0468
		MAS	-/13/-	0.9739	1.7263
		SG	-/14/-	0.9823	1.5239

Chrysin	SPA-PLS	Raw	4/5/-	0.6686	7.0933
		De-trending	7/9/-	0.8951	4.4475
		S.G	11/19/-	0.8743	5.1252

	SPA-LS-SVM	Raw	-/5/(2.66×10^3^,0.0074)	0.2867	9.1218
		De-trending	-/9/(0.0022,5.8750)	0.8450	9.3474
		S.G	-/19/(0.0667,1.59×10^3^)	0.6769	9.3138

	SPA-MLR	Raw	-/5/-	0.6774	6.9974
		De-trending	-/9/-	0.8919	4.5041
		S.G	-/19/-	0.8900	4.7457

	SPA-BP-ANN	Raw	-/5/-	0.9355	3.3515
		De-trending	-/9/-	0.9597	2.8953
		S.G	-/19/-	0.9668	2.4841
